# Functional diversification *of sonic hedgehog *paralog enhancers identified by phylogenomic reconstruction

**DOI:** 10.1186/gb-2007-8-6-r106

**Published:** 2007-06-08

**Authors:** Yavor Hadzhiev, Michael Lang, Raymond Ertzer, Axel Meyer, Uwe Strähle, Ferenc Müller

**Affiliations:** 1 Laboratory of Developmental Transcription Regulation, Institute of Toxicology and Genetics, Forschungszentrum Karlsruhe, Karlsruhe D-76021, Germany; 2 Laboratory of Developmental Neurobiology and Genetics, Institute of Toxicology and Genetics, Forschungszentrum Karlsruhe, Karlsruhe D-76021, Germany; 3 Department of Zoology and Evolution biology, Faculty of Biology, University of Konstanz, Konstanz D-78457, Germany; 4 Departament de Genètica, Universitat de Barcelona, Av. Diagonal 645, 08028 Barcelona, Spain

## Abstract

**Background:**

*Cis*-regulatory modules of developmental genes are targets of evolutionary changes that underlie the morphologic diversity of animals. Little is known about the 'grammar' of interactions between transcription factors and *cis*-regulatory modules and therefore about the molecular mechanisms that underlie changes in these modules, particularly after gene and genome duplications. We investigated the *ar-C *midline enhancer of *sonic hedgehog *(*shh*) orthologs and paralogs from distantly related vertebrate lineages, from fish to human, including the basal vertebrate *Latimeria menadoensis*.

**Results:**

We demonstrate that the *sonic hedgehog a *(*shha*) paralogs *sonic hedgehog b *(*tiggy winkle hedgehog*; *shhb*) genes of fishes have a modified *ar-C *enhancer, which specifies a diverged function at the embryonic midline. We have identified several conserved motifs that are indicative of putative transcription factor binding sites by local alignment of *ar-C *enhancers of numerous vertebrate sequences. To trace the evolutionary changes among paralog enhancers, phylogenomic reconstruction was carried out and lineage-specific motif changes were identified. The relation between motif composition and observed developmental differences was evaluated through transgenic functional analyses. Altering and exchanging motifs between paralog enhancers resulted in reversal of enhancer specificity in the floor plate and notochord. A model reconstructing enhancer divergence during vertebrate evolution was developed.

**Conclusion:**

Our model suggests that the identified motifs of the *ar-C *enhancer function as binary switches that are responsible for specific activity between midline tissues, and that these motifs are adjusted during functional diversification of paralogs. The unraveled motif changes can also account for the complex interpretation of activator and repressor input signals within a single enhancer.

## Abstract 2

Investigation of the *ar-C *midline enhancer of *sonic hedgehog *orthologs and paralogs from distantly related vertebrate lineages identified lineage-specific motif changes; exchanging motifs between paralog enhancers resulted in the reversal of enhancer specificity.

## Background

Phylogenetic footprinting can predict conserved *cis*-regulatory modules (CRMs) of genes that span over a number of transcription factor binding sites. However, divergence in sequence and function of CRMs over large evolutionary distances may hinder the utility of phylogenetic footprinting methodology [1-5]. Therefore, it is paramount also to investigate functionally the molecular mechanisms that underlie the function and divergence of CRMs. A vexing problem in elucidating the evolution of CRMs is that only a relatively small number of enhancers and other CRMs have thus far been characterized in sufficient detail to allow development of more general rules about their conserved structures and evolutionarily permitted modifications.

It is widely accepted that gene duplication is a major source for the evolution of novel gene function, resulting ultimately in increased organismal complexity and speciation [6-9]. It has been speculated that the mechanism by which duplicated genes are retained involves evolution of new expression times or sites through changes in their regulatory control elements [10-14]. An elaborate alternative model, called duplication-degeneration-complementation (DDC), has been proposed by Force and coworkers [15] to explain the retention of duplicated paralogs that occurs during evolution. Their model is based on the (often) multifunctional nature of genes, which is reflected by the multitude of regulatory elements specific to a particular expression domain. Mutations in subsets of regulatory elements in either one of the duplicated paralogs may result in postduplication spatial and temporal partitioning of expression patterns (subfunctionalization) between them. As a result, both paralogs can fulfil only a subset of complementary functions of the ancestral gene, and will thus be retained by selection and not be lost secondarily (for review [16]).

The diversity of possible mechanisms of subfunctionalization at the level of regulatory elements, however, is still poorly understood because of the lack of thorough comparative molecular evolutionary studies on *cis*-acting elements [2], supported by experimental verification of their function. Despite numerous presumed examples of subfunctionalization of gene expression patterns between paralogs, only two, very recent reports have included the necessary experimental verification of the hypothesis of subfunctionalization due to changes in CRMs [17,18]. Several studies, however, have implicated specific mutations in enhancers of parologous gene copies to be the likely source of subfunctionalization in duplicated *hox2b*, *hoxb3a*, and *hoxb4a *enhancers in fish [19-21].

Here, we report on an investigation into the molecular mechanisms of paralog divergence at the CRM level through the study of the duplicated *shh *genes in various lineages of 'fish', including *Latimeria menadoensis*. Teleost fish are well suited for analysis of *cis*-regulatory evolution in vertebrates [22,23]. Several teleost genomes have been sequenced, including those of the green spotted pufferfish (*Tetraodon nigroviridis*), fugu (*Takifugu rubripes*), zebrafish (*Danio rerio*), medaka (*Oryzias latipes*), and stickleback (*Gasterosteus aculeatus*). Adding them to the many available mammalian and anamniote vertebrate genomes covers a time span of 450 million years of evolution at different levels of genic and genomic divergence. More importantly, gene regulatory elements isolated from fish are suitable for functionality testing by transgenic analysis in well established model species such as zebrafish. Aside from conventional transgenic lines [24], CRMs can also be efficiently assayed directly in microinjected transient transgenic fish by analysis of mosaic expression through reporter activity [25-29]. Conserved sequences between mammals and Japanese pufferfish were first suggested to allow for predictions regarding the location of regulatory sequence [30-33]. This approach, combined with transgenic functional analysis, has allowed large-scale enhancer screening technologies to be applied in zebrafish [34-36].

The evolutionary history of the *hedgehog *gene family is well understood [37], and its biologic role has been extensively studied [38,39]. Comparative studies on the evolution of the vertebrate *hedgehog *gene family [37,40] showed that two rounds of duplication led to the evolution of three copies from a single ancestral *hedgehog *gene: *sonic hedgehog *(*shh*), *indian hedgehog *(*ihh*), and *desert hedgehog *(*dhh*). Several lines of evidence indicate that a complete genome duplication occurred early in the evolution of actinopterygian (ray-finned) fishes [41-46], leading to a large number of duplicated copies of nonallelic genes being found in different groups of teleosts [47-50]. Thus duplication of *shh *in the fish lineages resulted in two parlogous genes, namely *shha *and *shhb *[37,40], as well as duplication of *ihh *[51] and probably *dhh *genes as well.

The genes *shha *and *shhb *are both expressed in the midline of the zebrafish embryo [52]. There are, however, distinct differences between midline expression of the two paralogous genes, which may have important implications for their cooperative function. Although *shha *is expressed in the floor plate and the notochord, *shhb *is present only in the floor plate. Etheridge and coworkers [53] have shown that *shha *is expressed in notochord precursors and *shhb *is exclusively expressed in the overlying floor plate cells during gastrulation. Later, *shha *is expressed both in the notochord and floor plate, whereas *shhb *remains restricted to the floor plate [52]. The protein activity of *shhb *is very similar to that of *shha *[54]. It is likely that the concerted actions of *shha *and *shhb *are regulated quantitatively by their partially overlapping and tightly controlled level of expression. Thus far, the function only of *shha *has been studied in genetic mutants [55]. Nevertheless, morpholino knock-down and gene expression analyses identified several functions of the *shhb *gene. The *shhb *gene was shown to cooperate with *shha *in the midline to specify branchiomotor neurons, in somite patterning, but it is also required in the zona limitans intrathalamica and was implicated in eye morphogenesis [56-60].

The genomic locus of the zebrafish *sonic hedgehog a *gene is well characterized, and a substantial amount of data on the functionality of its *cis*-acting elements exist [26,61,62]. Enhancers that drive expression in the ventral neural tube and notochord of the developing embryo reside in the two introns and upstream sequences of both zebrafish and mouse *shh(a) *genes [26,63]. Comparison of genomic sequences between zebrafish and mammals in an effort to identify functional regulatory elements has verified the enhancers detected initially by transgenic analysis [23,64,65]. The conserved zebrafish enhancer *ar-C *directs mainly notochord and weak floor plate expression in zebrafish embryos [26,62]. This zebrafish enhancer also functions in the midline of mouse embryos [26], suggesting that the *cis*-regulatory mechanisms involved in regulating *shh(a) *expression are at least in part conserved between zebrafish and mouse. However, the mouse enhancer, SFPE2 (sonic floor plate enhancer 2), which exhibits sequence similarity with *ar-C *of zebrafish, is floor plate specific [63,66] and exhibits notochord activity only in a multimerized and truncated form [66]. This difference in enhancer activity emphasizes the importance of addressing the mechanisms of divergence in enhancer function between distantly related vertebrates. Given the observations on the *ar-C *enhancer in fish and mouse, we postulated that this enhancer might have been a target of enhancer divergence between *shha *and *shhb *paralogs in zebrafish during evolution.

Here, we show that a functional *ar-C *homolog exists in the *shha *paralog *shhb*. *Shhb ar-C *is diverged in function and became predominantly floor plate specific, similar to what has been found in the mouse *ar-C *homolog SFPE2. By phylogenetic reconstruction, we were able to predict the motifs that are required for the tissue-specific activity of the paralog enhancers, and we identified the putative transcription factor binding sites that were the likely targets of evolutionary changes underlying the functional divergence of the two *ar-C *enhancers of the *shh *paralogs. By engineering and exchanging mutations in both of the enhancers of *shha *and *shhb*, followed by transgenic analysis of the mutated enhancers, we were able to recapitulate the predicted evolutionary events and thus provide evidence for the likely mechanism of enhancer evolution after gene duplication.

## Results

### Selective divergence of *shhb *non-coding sequences from *shh(a) *genes

**Figure 1:**
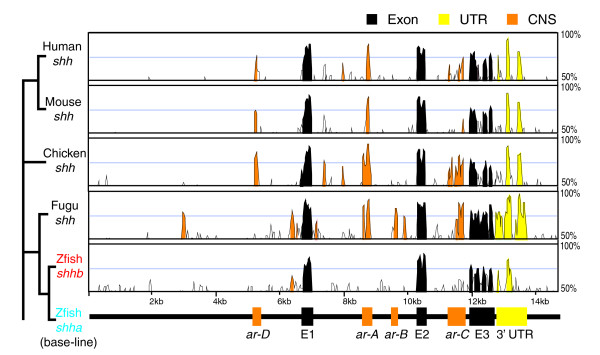
Selective divergence of *shhb *noncoding sequences from those of *shh(a) *genes. Vista plot of Shuffle-Lagan alignment of *sonic hedgehog (a) *(*shha*) and *sonic hedgehog b *(*shhb*) gene loci from different vertebrate species. The zebrafish *shha *locus is the base sequence with which the other *hedgehog*'s loci are compared. The peaks with more than 70% identity in a 50 base pair window are highlighted in color (color legend at the top). At the bottom of the plot, a scheme of the zebrafish *shha *locus marks the position of the exons, known *cis*-regulatory elements, and the 3'-untranslated region (UTR). The phylogenetic tree on the left side of the plot represents the evolutionary relationship of vertebrates. ar, activation region; CNS, conserved noncoding sequence; E, exon; kb, kilobase; UTR, untranslated region; zfish, zebrafish.

Comparisons of multiple vertebrate *shh *loci indicate a high degree of sequence similarity between zebrafish, fugu, chick, mouse, and human (Figure 1). A global alignment using shuffle Lagan algorithm and visualization by VISTA plot clearly identifies all three exons of *shh *orthologs and paralogs throughout vertebrate evolution (Figure 1). The CRMs identified previously are conserved among *shh(a) *genes (orange peaks), and the degree of their conservation is in accordance with the evolutionary distance between the species compared. In contrast, the zebrafish *shhb *gene exhibits no obvious conservation with the *shha ar-A*, *ar-B*, *ar-C*, and *ar-D *CRMs. Apart from Shuffle Lagan, Valis [36] has also failed to detect conserved putative CRMs of *shhb *(data not shown). Taken together, these findings indicate that although orthologous regulatory elements may exist between *shhb *and *shha*, they are much less conserved at the DNA sequence level than are *shha *elements, as detected by the applied alignment programs.

### The *ar-C *enhancer is a highly conserved midline enhancer of vertebrate *shh(a) *genes

**Figure 2:**
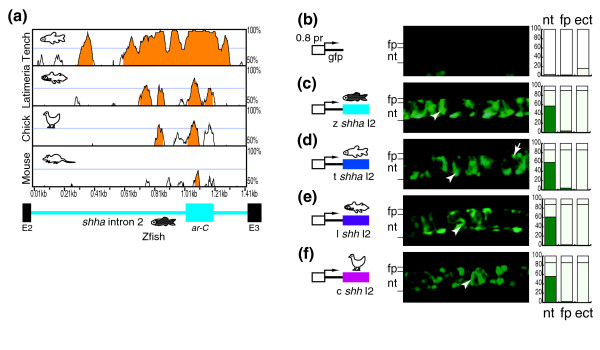
Vertebrate *ar-C *homolog enhancers function in the midline of zebrafish. **(a) **Vista plot comparison (AVID global sequence alignment algorithm) of *shha *intron 2 from zebrafish (base line), mouse, chick, *Latimeria*, and tench (bottom to top). The peaks showing more than 70% identity in a 50 base pair window are highlighted in orange. The scheme of the zebrafish *shha *intron 2 on the bottom marks the position of the zebrafish *ar-C *(blue rectangle), and the second and third exons (black rectangles). The remaining panels show a transgenic analysis of *shh intron 2 *fragments from vertebrates. Microinjected embryos are shown at 24 high-power fields with lateral view onto the trunk at the level of the midline. **(b) **Zebrafish embryo injected with control gfp-reporter construct, containing a minimal 0.8 kilobase zebrafish *shha *promoter. Also shown are embryos injected with gfp-reporter construct containing *shh(a) *intron 2 sequences from **(c) **zebrafish, **(d) **tench, **(e) ***Latimeria*, and **(f) **chick. The lines on the left side of each image mark the level of the notochord and the floor plate. The arrows point to floor plate cells and the arrowheads to notochord cells. The stacked-column graphs on the right side represent the quantification of the transient gfp expression. The columns show the percentage of the embryos with more than 15 green fluorescent protein (GFP)-positive cells per embryo (dark green), embryos with fewer than 15 cells (light-green), and nonexpressing embryos (white). Numbers of injected embryos are given in Table 1. ar, activation region; c, chick; E, exon; ect, ectopic; fp, floor plate; I, intron; k, kilobase; l, *Latimeria*; m, mouse; nt, notochord; pr, promoter; t, tench; z, zebrafish.

To characterize individual regulatory elements better, we focused on a single enhancer element *ar-C*, which is conserved between fish and mouse (SFPE2) and which has been analyzed in considerable detail in both species [26,63,66]. To this end, first we addressed whether the *ar-C *enhancer or its mouse ortholog SFPE2 is detectable across *shh(a) *loci in various vertebrate species from different lineages that diverged before and after the gene duplication event leading to the evolution of *shh *paralogs in zebrafish. Because the zebrafish *shha ar-C *enhancer is located in the second intron of *shha *and exhibits high sequence similarity to human and mouse counterparts, candidate *ar-C *containing intronic fragments of several vertebrate species were amplified by polymerase chain reaction (PCR) with degenerate oligonucleotide primers. We cloned and sequenced the relevant genomic DNA fragments from several fish species that experienced the genome duplication, such as the cyprinid tench (*Tinca tinca*), fugu, and medaka [45]. In addition to actinopterygian fishes, several species of sarcopterygians such as chick, mouse, and the early sarcopterygian lineage *Latimeria menadoensis *were used in the analysis. All sarcopterygians diverged from the common ancestor with actinopterygians before the fish-specific genome duplication in the ray-finned fish lineage. A sequence comparison of intron 2 sequences from the available vertebrate model systems revealed a high degree of sequence similarity in all species specifically in the region that spans the *ar-C *enhancer in zebrafish and the SFPE2 enhancers of mouse (Figure 2a). This analysis also indicated that the orthologous *Latimeria *genomic region also contains a highly conserved stretch of sequence in the *ar-C *region, which is consistent with the hypothesis that *ar-C *is an ancestral enhancer of *shh *genes.

### Heterologous *ar-C *enhancers function in the notochord of zebrafish

To test whether the sequence similarity observed between *ar-C *enhancers of different lineages of vertebrates is also indicative of conserved tissue-specific enhancer function, we carried out transgenic analysis of enhancer activity in microinjected zebrafish embryos. We utilized a minimal promoter construct (containing an 0.8 kilobase [kb] upstream sequence from the transcriptional start site with activity similar to the -563*shha *promoter described by Chang and coworkers [67], linked to green fluorescent protein (GFP) reporter. Transient mosaic expression of GFP was measured as read-out of reporter construct activity by counting fluorescence-positive cells in the notochord and floor plate, where the *ar-C *enhancer is active, in the trunk of 1-day-old embryo (Table 1). This approach was a reliable substitute for the generation of stable transgenic lines, as reflected by the identical results obtained with transient analysis and stable transgenic lines made for a subset of the constructs used in this study (Additional data file 1).

**Table 1:** Quantification of GFP expression for each reporter construct

Reporter construct	Notochord >15 cells	Notochord <15 cells	Floor plate >15 cells	Floor plate <15 cells	Ectopic >15 cells	Ectopic <15 cells	Nonexpressing	Total number
*0.8shha:gfp*	0%	3 ± 1.6%	0%	2.3 ± 0.9%	0%	16 ± 3.5%	84 ± 3.5%	224
*0.8shha:gfp:z-shha-I2*	57 ± 2.9%	32.9 ± 5.2%	3.4 ± 1.2%	86.5 ± 3%	0%	89.9 ± 3.8%	10.1 ± 4.7%	301
*0.8shha:gfp:t-shha-I2*	58.8 ± 3.3%	27.1 ± 6.7%	4 ± 0.7%	82 ± 4.6%	0%	86 ± 4%	14 ± 4.9%	272
*0.8shha:gfp:l-shh-I2*	61.2 ± 8.5%	26.4 ± 5.2%	1.2 ± 0.3%	86.4 ± 3.5%	0%	87.6 ± 3.4%	12.4 ± 4.2%	325
*0.8shha:gfp:c-shh-I2*	56.1 ± 7.2%	28.9 ± 11.5%	2 ± 0.1%	83.1 ± 4.2%	0%	85 ± 4.3%	15 ± 6.1%	203
*0.8shha:gfp:z-shhb-I2*	30.2 ± 5.3%	51.6 ± 6.9%	38.1 ± 4.9%	43.7 ± 8.9%	2.5 ± 1%	79.3 ± 4.6%	18.2 ± 5.4%	281
*0.8shha:gfp:t-shhb-I2*	27.9 ± 7.9%	50.9 ± 7.9%	37.8 ± 5.7%	41 ± 6%	2.1 ± 0.7%	76.8 ± 2%	21.2 ± 3.3%	248
*0.8shha:gfp:z-shhb-I2-non.cons.*	0%	1.3 ± 1.3%	0%	2.1 ± 0.8%	0%	7.7 ± 2.4%	92.3 ± 3.5%	145
*0.8shha:gfp:z-shhb-arC*	36.7 ± 5.7%	48.9 ± 7.2%	46 ± 5.4%	39.6 ± 10.3%	3.1 ± 0.3%	82.4 ± 4.8%	14.4 ± 6.9%	409
*0.8shha:gfp:z-shha-arC*	62.2 ± 5.6%	28.6 ± 2.4%	4.4 ± 1.1%	86.4 ± 3.4%	0%	90.8 ± 3.5%	9.2 ± 4.3%	260
*0.8shha:gfp:z-shha-arCΔ*C1	0%	2.2 ± 0.6%	0%	1.5 ± 0.1%	5.2 ± 0.3%	11.9 ± 1%	82.9 ± 0.9%	135
*0.8shha:gfp:z-shha-arCΔ*C2	46.2 ± 4.3%	31.1 ± 8.8%	5 ± 1.3%	72.2 ± 3.3%	0%	77.2 ± 4.5%	22.8 ± 5.6%	347
*0.8shha:gfp:z-shha-arCΔ*C3	51.2 ± 3.6%	30.5 ± 2.6%	47.1 ± 4.5%	34.6 ± 5.7%	3.7 ± 1.3%	78 ± 1.9%	18.3 ± 3.7%	307
*0.8shha:gfp:z-shha-arCΔ*C4	32.5 ± 5.1%	48.6 ± 6.6%	37.6 ± 3.1%	43.5 ± 4.8%	2.1 ± 1.3%	79.1 ± 5%	18.9 ± 4.7%	359
*0.8shha:gfp:z-shha-arC+*C4m	36.8 ± 6.2%	41.6 ± 5.4%	42.3 ± 7.2%	36.1 ± 6.7%	2.8 ± 1.6%	75.6 ± 4.5%	21.6 ± 5.1%	174
*0.8shha:gfp:z-shhb-arCΔ*C1	0%	0%	0%	0%	3.8 ± 1.6%	10.7 ± 7.7%	85.5 ± 11.3%	186
*0.8shha:gfp:z-shhb-arCΔ*C3	33.5 ± 3%	40.5 ± 6%	37.8 ± 3.9%	36.2 ± 7.3%	0%	74 ± 3.5%	26 ± 4.3%	230
*0.8shha:gfp:z-shhb-arC*+C2	23 ± 6.2%	44.6 ± 8.7%	36 ± 5.2%	31.6 ± 7.8%	1.3 ± 1%	66.3 ± 3.2%	32.4 ± 3.2%	203
*0.8shha:gfp:z-shhb-arC*+C4	45.7 ± 7.2%	43.3 ± 4.7%	8.2 ± 2.4%	80.8 ± 3.8%	0%	89 ± 3.2%	11 ± 3.9%	288
*2.7shhb:gfp*	72.4 ± 3.1%	19.6 ± 3.3%	0%	92 ± 3.4%	0%	92 ± 3.4%	8 ± 4.2%	308
*2.7shhb:gfp:z-shhbI1*	68 ± 4.9%	19.8 ± 0.8%	0%	87.8 ± 4.2%	0%	87.8 ± 4.2%	12.2 ± 5.1%	339
*2.7shhb:gfp:z-shhbI2*	61.4 ± 4.9%	24.7 ± 2.9%	36.4 ± 3.6%	49.7 ± 3.1%	2 ± 0.8%	84.1 ± 5.6%	13.9 ± 7.7%	296

Values are expressed as mean ± standard deviation. GFP, green fluorescent protein.

As described previously, the zebrafish *ar-C *enhancer is primarily active in the notochord and only weakly in the floor plate (Figure 2c). Intron 2 sequences of tench, chick, and *Latimeria shh *genes gave strong enhancer activity in the notochord (Figure 2d-f). However, the mouse intron 2 (with the SFPE2 enhancer) was found to be inactive in zebrafish (data not shown), suggesting that SFPE2 had functionally diverged during mammalian/mouse evolution either at the *cis*-regulatory or the *trans*-regulatory level. All together, these data indicate a high degree of functional conservation between *ar-C *sequences among vertebrates.

### Identification of a putative *ar-C *enhancer from *shhb *genes

The evolutionary functional divergence of paralogous *ar-C *enhancers was tested through the isolation of the *shhb *intron 2 from zebrafish. Because a genome duplication event has taken place early in actinopterygian evolution, it was predicted that the ostariophysian and cyprinid zebrafish as well as all acanthopterygian fish model species whose genomes are known (medaka, stickleback, green spotted pufferfish, and fugu) may contain a *shhb *homolog. Analysis of the available genome sequences of these four species of teleost fish indicated that none of them carries a discernible *shhb *homolog, suggesting that these lineages (which evolved some 290 million years after cyprinids [68]) may have secondarily lost this *shh *paralog. Synteny is observed between the medaka genomic region surrounding *shh *on chromosome 20 and a region on chromosome 17; however, chromosome 17 lacks *shhb *(Additional data file 2). This finding further supports the hypothesis that a *shhb *gene was originally present after duplication but has been lost secondarily during evolution.

**Figure 3:**
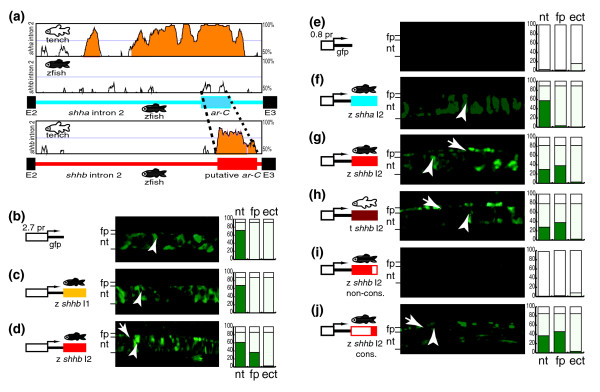
*Shhb *genes carry a functional *ar-C *homolog enhancer with diverged sequence and tissue specificity. **(a) **Top panel: Vista plot comparison (AVID) between zebrafish *shha *intron 2 (baseline), zebrafish *shhb *intron 2, and tench *shha *intron 2. Bottom panel: comparison between zebrafish (baseline) and tench *shhb *intron 2. The peaks exhibiting more than 70% identity in a 50 base pair window are highlighted in orange. The schemes of zebrafish *shha *(top) and *shhb *(bottom) intron 2 mark the position of the *shha ar-C *(blue box), the putative *shhb ar-C *(red box), and exons *2 *and *3 *(black boxes). Dashed lines demarcate equivalent sequence regions. Panels b to d show a transgenic analysis of *shhb *genomic fragments for enhancer activity. Embryos injected with the plasmid constructs are shown at 24 high-power field (hpf), lateral view, onto the trunk at the level of midline. Shown are embryos injected with gfp-reporter constructs containing zebrafish **(b) **2.7 kilobase (kb) *shhb *promoter, **(c) **2.7 kb *shhb *promoter plus zebrafish *shhb *intron 1, and **(d) ***shhb *intron 2. Panels e to j show transgenic analysis of the enhancer activity of *shha *and *shhb *intron 2 fragments. Shown are embryos injected with **(e) **promoter-control construct, **(f) **plasmids containing zebrafish *shha *intron 2, **(g) **zebrafish *shhb *intron 2, **(h) **tench *shhb *intron 2, **(i) **the nonconserved part of zebrafish *shhb *intron 2, and **(j) **the conserved part (putative *ar-C*). Arrows and arrowheads indicate green fluorescent protein (GFP) activity in the floor plate and notochord cells, respectively. Lines on the left side indicate the level of the floor plate and notochord on the images. The quantification of the gfp expression is shown on the graphs, as described above. ar, activation region; E, exon; ect, ectopic; fp, floor plate; I, intron; nt, notochord; pr, promoter; t, tench; z, zebrafish.

However, we were able to detect and isolate *shhb *and its intron 2 from another cyprinid species, tench, by PCR using degenerate oligonucleotides that were designed in conserved exon sequences. Importantly, the isolation of more than one *shhb *intron 2 sequences from cyprinids allowed for phylogenetic footprinting of *shhb *genes and a search for a putative *ar-C *homolog. We have compared the *shha *and *shhb *intron 2 sequences between zebrafish and tench (Figure 3a). The *shha *orthologs between zebrafish and tench exhibit a high degree of sequence similarity, which is strongest in the region in which *ar-C *resides. In contrast, comparison of intron 2 from *shhb *and *shha *paralogs of either species revealed no conspicuous conservation. The apparent lack of sequence similarity, however, does not necessarily rule out the possibility that a highly diverged *ar-C *homolog enhancer may still reside in *shhb *intron 2. A sequence comparison between zebrafish and tench *shhb *intron 2 reveals a striking sequence similarity in the 3' region close to exon 3, where a positionally conserved *ar-C *would be predicted to be located. This suggests that intron 2 of *shhb *genes of cyprinids may contain a functional enhancer, which has diverged significantly from the *shha ar-C*. Furthermore, the apparent sequence divergence suggests that the function of the *shhb *enhancer may also have diverged.

### The diverged *ar-C *enhancer of *shhb *is functionally active

To test whether the conserved sequence in the intron 2 of *shhb *genes is indeed a putative enhancer element, we tested several *shhb *fragments representing approximately 10 kb of the locus in transgenic reporter assays. The *shhb *proximal promoter and 2.7 kb of upstream sequences can activate GFP expression in the notochord (Figure 3b) but only very weakly in the floor plate, similarly to previously reported data [69]. Because *shhb *is only expressed in the floor plate and never in the notochord, this GFP expression of the reporter is an ectopic activity and reflects the lack of a notochord repressing functional element, probably located elsewhere in the unexplored sequences around the *shhb *locus. The weak expression in the floor plate suggests that other CRMs are required for floor plate activation. In *shha *a floor plate enhancer resides in intron 1 [26]. To check whether a similar enhancer exists in *shhb*, intron 1 of *shhb *was attached to the promoter construct. It was found that it did not enhance the promoter's activity, indicating no obvious enhancer function in this transgenic context (Figure 3c). Interestingly, the addition of *shhb *intron 2 does result in enhancement of expression in the floor plate (Figure 3d). This finding indicates that intron 2 of *shhb *contains a floor plate enhancer.

The 2.7 kb upstream and proximal promoter sequence of *shhb *may have influenced the autonomous function of an enhancer in intron 2. To address the activator functions of the identified *shha *and *shhb *enhancers without influence of potential upstream regulatory elements, a series of injection experiments was carried out in which the enhancer activities were analyzed with a minimal promoter containing only 0.8 kb of the *shha *promoter (Figure 3e-j). Moreover, activity of intron 2 sequences from *shha *and *shhb *genes from both zebrafish and tench were systematically compared. *Shha *intron 2 fragments of both species consistently resulted in comparable notochord activity (Figure 3f and Additional data file 1 [parts B and C]), wheres the *shhb *intron 2 fragment from both species exhibited distinct enhancement of expression in the floor plate and reduction in GFP activity in the notochord (Figure 3g,h). The presence of a highly conserved region within the intron 2 of zebrafish and tench *shhb *genes strongly suggests that the floor plate enhancer activity is the property of this conserved sequence. To test this prediction a set of deletion analysis experiments was carried out. Zebrafish *shhb *intron 2 was cleaved into a 1,026 base pair (bp) fragment of nonconserved and a 380 bp conserved sequence. As shown in Figure 3i,j, the floor plate specific enhancer effect is retained by the conserved fragment but not by the non-conserved sequence, verifying the prediction of the location of the floor plate enhancer. Taken together, a diverged, floor plate active *ar-C *enhancer has been discovered in the *shhb *intron 2, which is consistent with the floor plate specific expression of *shhb *in zebrafish.

### Prediction of functionally relevant motifs by phylogenetic reconstruction

**Figure 4:**
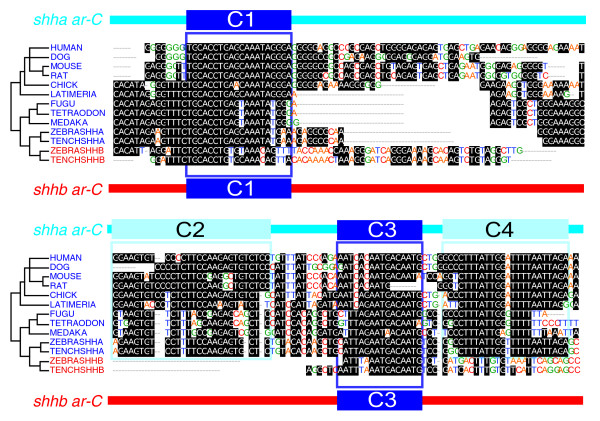
Sequence comparison identifies phylogeny-specific, paralogy-specific, and orthology-specific conserved motifs in *ar-C *sequences. Multiple alignment of *ar-C *homolog sequences of *shh(a) *and *shhb *genes of different vertebrate species was carried out. The phylogenetic tree on the left side represents the evolutionary relationship of the vertebrates. Species in blue correspond to *ar-C *of *shh(a) *genes, and those in red to *ar-C *of *shhb *genes. Dark blue boxes depict the conserved motifs, present in both *shh(a) *and *shhb ar-C *genes. Light-blue boxes mark motifs present only in *shhb *genes.

Transcription factor binding sites may be more conserved than the surrounding sequences [70]. We have hypothesized that sequence similarity between fish and human *ar-C *sequences may indicate conserved motifs, which may reflect conserved transcription factor binding sites [66]. We postulated that putative transcription factor binding sites and changes in them may be detectable by identification of motifs using local alignment of *ar-C *from large numbers of pre-duplicated and post-duplicated *shh *orthologs and paralogs. To this end, a CHAOS/DIALIGN [71] alignment was used to compare the functionally active *ar-C *enhancer of zebrafish (as described by Muller and coworkers [26]) and equivalent sequences from all major vertebrate classes. The alignments were arranged according to phylogeny (Figure 4).

A pattern of conserved motifs is detected in the form of homology blocks extending to 20 to 30 bp. These conserved motifs exhibit distinct distribution characteristics, which reflect phylogenic as well as paralogy and orthology relationships between *shh *genes. C1 and C3 are homology blocks, which are present in all *shh *sequences, including *shhb *paralogs, in all species analyzed. In contrast, C2 and C4 are homology blocks that are present only in *shh(a) *genes but absent in *shhb *genes. Because C2 and C4 are present in pre-duplicated enhancers of sarcopterygians, the lack of C2 and C4 in *shhb *enhancers is probably due to a secondary loss of these elements after the fish-specific gene duplication. The two sets of putative binding sites (C1/C3 and C2/C4, respectively) may thus be targets for transcription factors that regulate the differential enhancer activities of *shh(a) *(predominantly notochord expression) and *shhb *(predominant floor plate expression). In conclusion, we identified a set of putative targets of mutations that may contribute to the divergence of *ar-C *enhancer functions after gene duplication.

### Functional analysis of conserved motifs reveals the evolutionary changes that likely contributed to the enhancer divergence of *shh *paralogs

To test the functional significance of the two sets of homology blocks, we conducted a systematic mutation analysis of the C1 to C4 conserved homology blocks in both *shha *and *shhb *genes. Furthermore, we carried out exchange of homology blocks between *shha *and *shhb ar-C *enhancers to test whether evolutionary changes after gene duplication can be modeled in a transgenic zebrafish system.

**Figure 5:**
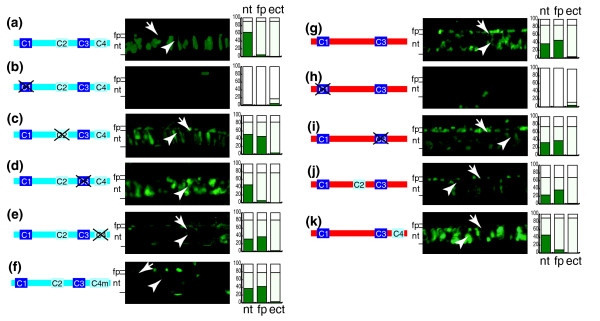
Functional analysis of *shha *and *shhb ar-C *conserved motifs. This analysis reveals the basis for divergence in tissue specificity. Panels a to e show a transgenic analysis of *shha ar-C *motifs by site specific mutations. Embryos injected with the corresponding constructs are shown at 24 hours post-fertilization (hpf) lateral view onto the trunk at the level of the midline. Shown are embryos injected with gfp-reporter constructs containing **(a) **wild-type zebrafish *shha ar-C*, **(b) ***ar-C *with mutated C1 region, **(c) **mutated C2, **(d) **mutated C3, **(e) **mutated C4, and **(f) **C4 replaced with medaka C4 (C4m). Panels g to k show a transgenic analysis of *shhb ar-C *motifs. Shown are embryos injected with gfp-reporter constructs containing **(g) **wild-type zebrafish *shhb ar-C*, **(h) ***ar-C *with mutated C1 and **(i) **mutated C3, and with **(j) **exchange of *shhb *sequence with the zebrafish *shha *C2 and with **(k) **the zebrafish *shha *C4. Stacked-column graphs show the quantification of the gfp expression, as described in Figure 3. Arrows and arrowheads point to floor plate and notochord cells, respectively. Lines on the left side indicate the level of the floor plate and notochord on the images. ect, ectopic; fp, floor plate; nt, notochord.

As shown in Figure 5b-f, mutations inserted into homology blocks (C1 to C4) result in dramatic changes in *shha ar-C *enhancer activity. Replacement of C1 with random sequence results in total loss of *ar-C *enhancer function, indicating that this binding site is critical for *shha ar-C *activity (Figure 5b). By contrast, loss of C3 results in no observable effect, suggesting that this conserved block is either not required for enhancer function or only necessary for functions that are not detectable in our transgenic system (Figure 5d). Importantly, removal of C2 or C4 (the blocks that are only present in *shha *genes) results in strong expression of GFP in the floor plate (Figure 5c,e). In the case of C4 removal, a reduced reporter expression in the notochord has also been observed (Figure 5e). The obtained expression pattern strongly resembles the activity of the wild type *shhb ar-C *enhancer (compare panels e and g of Figure 5). Thus, removal of *shha*-specific motifs from the *shha ar-C *mimics *shhb ar-C *enhancers. Moreover, this result is consistent with a model in which the C2 and C4 elements are targets for repressors of floor plate expression in the *shha ar-C *enhancer.

The multiple alignment of *ar-C *homolog sequences revealed a noticeable modification in the C4 element of acanthopterygian fishes, which do not have a *shh *paralog (fpr example, medaka and fugu; see Figure 4 and Additional data file 3 for alternative alignment results). The divergence in the C4 motif of acanthopterygians may reflect a functional change in the *ar-C *enhancer in these species, potentially leading to the relaxation of the floor plate repression observed in *ar-C *of *shha *genes. To test whether the modification of the C4 motif of acathopterygians may reflect the loss or modification of C4 repressor function, we have replaced the C4 of zebrafish *shha *with that of medaka *shh*. The resulting hybrid construct activated strong expression in the floor plate (Figure 5f), suggesting that the medaka C4 motif is unable to rescue the repressing activity of zebrafish *shha *C4 in zebrafish embryos.

We next asked whether *shhb ar-C *is active in the floor plate because it contains the general midline activator site C1 and lacks the floor plate repressor elements C2 and C4 that are present in the *shha ar-C *enhancer. To this end, we first tested whether the C1 and C3 of *shhb *are required for the function of the *shhb *enhancer. Similar to the results obtained with *shha*, C1 was found to be critical for the activity of *shhb ar-C *(compare panels b and h of Figure 5), whereas loss of C3 had no effect, thus mimicking the findings in *shha *(Figire 5i). We then introduced C2 or C4 into the *shhb *enhancer in order to test the functional significance of the lack of C2 and C4 motifs in *shhb*. When a *shh*-derived C2 was introduced into *shhb ar-C*, no effect was observed (Figure 5j), but introduction of the C4 putative floor plate repressor motif from *shha *did result in a dramatic shift in *shhb *enhancer activity (Figure 5k). The effect was a repression of floor plate expression while notochord activity was retained, thus resembling the wild-type or C2 mutant *shha ar-C *enhancer (Figure 5a,c). In a control experiment, random DNA sequence was introduced at similar positions into the *shhb ar-C *enhancer. However, this manipulation had no effect on the activity of *shhb ar-C *(data not shown), indicating that the changes observed with the C4 insertion are due to the specific sequence of C4. These results together strongly suggest that the function of C4 is to repress floor plate activation by the *shha *ar-C enhancer. Together, these findings are consistent with a model in which loss of the C4 motif in the evolution of the *shhb ar-C *has contributed to its floor plate specific activity.

## Discussion

It has long been suggested [72,73] that a major driving force in evolution of animal shape results from divergence of *cis*-regulatory elements of genes. Recent years have provided evidence in support of this hypothesis [11-13,74-76]. However, the mechanisms of regulatory evolution are still poorly understood [1,5,77,78]. In this report, we have systematically analyzed the evolutionary history of a single enhancer of orthologous and paralogous *shh *genes during vertebrate phylogeny. By constructing multiple alignments, we were able to predict which motifs within the *ar-C *enhancer represent regulatory input. Through specific mutations and exchanges of motifs, we mimicked probable evolutionary events in transgenic analysis and identified the lineage-specific modifications that lead to discernible changes in tissue-specific enhancer activity in embryo development.

### Identification and functional verification of a diverged *ar-C *enhancer

Using phylogenetic footprinting of intron 2 of *shhb *genes we have identified a conserved *ar-C *homolog enhancer in two species of cyprinids. The results of our transgenic analysis indicate that the *ar-C *sequences in intron 2, together with the promoter activity of *shhb *[69], contribute to this gene's activity in the floor plate. Although *shh(a) *enhancers retained significant sequence similarity with their orthologs, the whole of the *shhb *gene and its *ar-C *enhancer is grossly changed from that of *shha *paralogs. This paralog-specific change happened despite the fact that *shhb *had equal time and chance to diverge as did *shha *after duplication from an ancestral *sonic hedgehog *gene. This result is in accordance with observations indicating selective pressure on the CRMs of paralogs in invertebrates [79] as well as in vertebrates [19,20,80,81]. Our results, together with the reports cited above, provide experimental support to the notion that differential divergence of noncoding conserved elements of paralogs may be a general phenomenon in vertebrates [35].

### Identification of putative transcription factor binding sites by local alignment of multiple species

Use of a local sequence alignment approach of representative species of major vertebrate lineages allowed us to predict functionally relevant motifs within the *ar-C *enhancers. Our findings are most consistent with a model in which these motifs are individual or multimeric transcription factor binding sites. Mutation and transgenic analysis verified the functional relevance of these motifs in driving expression in the midline, and therefore the most parsimonious explanation for the conservation of these sequence elements is that they represent functional binding sites for developmental regulatory transcription factors.

**Figure 6:**
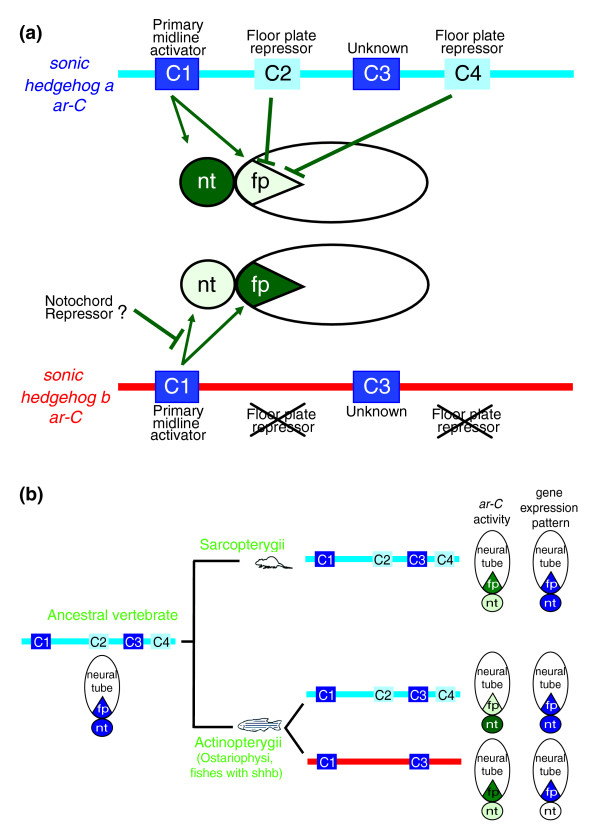
The mechanism of functional divergence of *ar-C *enhancers of duplicated *shh *genes in zebrafish. **(a) **Model for motif structure and interaction in *ar-C *enhancers involved in the regulation of midline expression of *shha *and *shhb *in zebrafish. Schemes on the top and bottom represent the structure of the *ar-C *enhancer of *shha *(blue) and *shhb *(red) with the position of the conserved motifs indicated in colored boxes, as in Figures 4 and 5. In the middle, schematic cross-sections of the neural tube with the floor plate (fp) and the notochord (nt) are shown (ventral to the left). Dark green indicates strong enhancer activity. Arrows indicate activator and blunt arrows indicate repression function by individual motifs. **(b) **Evolution of *ar-C *enhancers of vertebrates. Phylogenetic relationship of the genes and the motif composition of the respective *ar-C *enhancers are shown. *Shha *gene enhancers are shown in blue and *shhb *gene enhancers in red. On the left, a predicted pre-duplicated ancestral *shha ar-C *enhancer is shown. Below, the predicted activity of the ancestral *shha *gene is depicted in blue in a schematic cross-section of an embryonic midline. On the right, schematic cross sections of midlines in green indicate *ar-C *(SFPE2 [sonic floor plate enhancer 2]) enhancer activities; shades of green indicate strength of enhancer activity in the respective midline tissues. In blue the expression activity of the respective *shha*/*shhb *genes are shown.

The *ar-C *enhancer is composed of motifs with different regulatory capacities (Figure 6a). Motifs exist that are crucial for the overall activity of the enhancer (C1), whereas other repressor motifs refine enhancer activity (C2 and C4). This indicates that the overall activity output of an enhancer in midline tissues is subject to both activator and repressor functions acting in concert. These results are in accordance with the previously proposed grammar of developmentally regulated gene expression [11,82-87]. Importantly, the order and combination of motifs of *ar-C *are conserved. This is a very different result from that proposed for the stripe 2 enhancers of drosophilids, in which the functional conservation of CRMs was a result of stabilizing selection of reshuffled transcription factor binding site composition [1,77]. The evolutionary pressure to keep the order and composition of binding sites within enhancers may be limited to transcription factor and developmental regulatory genes [88,89]. The high conservation level, however, may be a consequence of selective pressure acting on a secondary function of enhancer sequences [90].

Previously, individual binding sites were identified through comparative approaches in vertebrates (for instance, see [66,91,92]). These examples, together with our systematic analysis of conserved motifs in the *ar-C *enhancers, demonstrate that functionally relevant motifs detected by sequence alignment may aid in identifying as yet unknown and uncharacterized functional transcription factor binding sites.

### Phylogenetic reconstruction of enhancer divergence at the level of conserved motifs

The use of large numbers of species spanning long evolutionary distances allowed us to generate a phylogenetic reconstruction of enhancer divergence before and after gene duplication (Figure 6b). By generating artificial enhancers with mutations that mimic the predicted lineage-specific changes in motif composition of *shhb *and *shha *enhancers, we were able to reconstruct the probable evolutionary events leading to divergence of the *ar-C *enhancer function. For example, insertion of the floor plate repressor C4 element into *shhb *resulted in enhancer activity reminiscent of *shha ar-C*, in which the C4 site had been identified. These findings indicate that the very changes that resulted in the divergence of the enhancer function have been identified.

An open question remains, however; why should the *ar-C *enhancer of *shha *be repressed in the floor plate while the *shha *gene is well known to be active in this tissue? The level of the Hedgehog morphogen signal emanating from the embryonic midline is critical for correct patterning of the ventral neural tube [93]. Animals with only one gene encoding the Sonic hedgehog protein (sarcopterygians and fishes without *shhb*) achieve this by controlled activation of *shh *in the notochord and floor plate as a result of a combination of several synergistic enhancers [62,63]. In zebrafish and other ostaryophisian species (for instance, tench and Mexican cavefish) a second copy of *shh *paralog (*shhb*) also contributes to Shh production in the floor plate. At least in zebrafish, controlled levels of the floor plate expressed *shhb *are required, together with the notochord and floor plate derived *shha*, for normal patterning of branchiomotor neurons and the somites [56-58]. The combined activity of two *shh *genes emerging from the floor plate and notochord may thus result in one of the paralog floor plate enhancers being subjected to selection pressure. For example, to counter the overproduction of Hedgehog levels, the reduction in transcription can occur by blocking the activity of one of the synergistically active enhancers (in this case *ar-C*). It is important to note, however, that the *shh(a) ar-C *enhancers are not exclusively expressed in the notochord, and retained a weaker but still noticeable capacity to activate expression in the floor plate. Thus, the output of Shh levels in zebrafish appears to be a subject of quantitative regulation of paralog enhancer activities. Alternatively, it is feasible that there are time points when the two paralog genes are not overlapping in expression and the complementing specificities of *shhb *and *shha ar-C *enhancers reflect the non-overlapping production of Hedgehog proteins in the two midline tissues [53].

### Subfunctionalization by fission or binary switch in midline specificity of enhancers during evolution

Recent reports have provided experimental verification of subfunctionalization of Hox gene enhancers [17,18]. Our report adds to those findings by contributing evidence for the diversity of subfunctionalization mechanisms that may act on paralog enhancers during evolution. Here, we propose that the presence or absence of the C4 site functions as a binary switch to modulate *ar-C *enhancer activity specific to one of two midline tissues after gene duplication. By selective removal of repressor and activator binding sites, subfunctionalization of the *ar-C *enhancer to floor plate or notochord can thus occur (Figure 6b). This model is reminiscent of those proposed for subfunctionalization of CRMs [15].

The subfunctionalization model would argue for the existence of a preduplication (sarcopterygian) *ar-C *enhancer that is equally active in both floor plate and notochord. Interestingly, the mouse *ar-C *homolog SFPE2 enhancer is mainly active in the floor plate of the mouse [63] and can activate notochord expression in a multimerized form [66] (Figure 6b). However, in fish all *shh ar-C *enhancers from sarcopterygian lineages exhibit notochord-specific enhancer activity. The differences between zebrafish and mouse may be explained both by subfunctionalization mechanisms as well as by *trans*-acting factor changes. In support of *trans *changes the mouse SFPE2 enhancer exhibited no activity in the fish. In the converse experiment, the mainly notochord-specific zebrafish *shha ar-C *exhibited both floor pate and notochord activity in mouse [26]. Thus, the subfunctionalization of duplicated *ar-C shh *enhancers is a composite result of selective loss of several motifs, including negative regulatory elements in one enhancer (*shhb*) paralleled by modifications either on the *cis *or on the *trans *level to restrict activity of the less diverged sister paralog enhancer (*shha*). The prediction from this model is that fish species without *shhb *gene (acantopterygii) may have floor plate active *ar-C *enhancer. Interestingly, the floor plate repressor elements (C2/C4) of *shha ar-C *of acanthopterygians (for example, medaka and fugu) are present but diverged from all other *shh(a) *homologs (Figure 4), and they may thus represent the evolutionary changes that lead to retention of *shh ar-C *floor plate activity in these fish lineages. Our experiments with the medaka *shh *C4 element replacing that of zebrafish *shha *provide further support to the model outlined above. The hybrid zebrafish *shha ar-C *construct with the modified medaka C4 motif cannot rescue the loss of the zebrafish *shha *C4 element and does not function as a repressor site in zebrafish. These findings are in line with a predicted compensatory relaxation of repressor function of *shh ar-C *in medaka.

The combination of both negative and positive regulatory sites within a single enhancer indicates the integration of activating and repressing signals to modulate the resulting transcriptional activity. This could be achieved through multiple *trans*-acting factors that interact with a series of binding sites within the *ar-C *enhancer. Determining which transcription factors bind to the C1 to C4 blocks remains a challenge for future research. Predictions can be made based on known transcription factor recognition sequences. For instance, C1 contains a *foxA2 *binding sequence, which is consistent with the previously suggested role of this factor in regulating *shh *gene expression in the midline of mouse [66,94], frog [95], and fish [67]. Interestingly, C4 carries a sequence identical to the homeobox binding site that has been described to be present in the mouse SFPE2 enhancer [66]. This binding site is required for floor plate activity in the mouse. The identity of the mouse binding factor and whether the same transcription factor acts (probably by repressing floor plate activity) in the *ar-C *enhancer in zebrafish are unknown. The relevance of specific transcription factors from large protein families in binding to the *ar-C *binding sites remains a challenging question. It is important to note, however, that the functionally relevant sequences in SFPE2 that are responsible for floor plate activity in the mouse (HR-c) [66] only partially overlap with *ar-C *sequences that are functionally relevant in zebrafish, and this divergence may explain, at least in part, the different results obtained with mouse and fish enhancers.

## Conclusion

In conclusion, the observed changes in the duplicated *shh ar-C *enhancers provided novel insights into the functional components of enhancer divergence in an important developmental regulator gene. In particular, our findings demonstrate that phylogenetic reconstruction using large number of vertebrate species can identify a series of lineage specific motifs that were the probable targets of evolutionary change and represent individual regulatory input acting in concert on a developmentally regulated gene enhancer. These findings reinforce the importance of the phylogenomic and functional analysis of duplicated *cis*-regulatory elements in deciphering the *cis*-regulatory code of developmental gene regulation.

## Materials and methods

### Isolation of *shh(a) *and *shhb *intron 2 sequences

The tench *shha *and *shhb *intron 2 fragments were isolated by using degenerative oligonucleotides, designed based on conserved amino acid blocks in the second and third exons of *shh(a) *and *shhb *genes from several vertebrate species. The PCR products were directly cloned into *pCRII-TOPO *vector (Invitrogen, Carlsbad, CA, USA), and the clone containing the right insert was identified by sequencing.

The *Latimeria *intron 2 was isolated by screening of genomic bacterial artificial chromosome (BAC) library from *Latimeria menadoensis *[96] (Lang and coworkers unpublished data), kindly provided by Chris Amemiya. The positive BAC clone, containing the *shh *locus, was shotgun sequenced and relevant genomic regions were secondarily amplified by gene specific primers. The correct PCR product was identified by sequencing. The mouse and chick intron 2 were directly amplified from genomic DNA with specific oligonucleotides.

### Plasmid construction

The *0.8shha:gfp *plasmid was constructed by cutting out the *Sal*I/*Hind*III fragment from *2.4shha:gfp *plasmid [62] (described as *2.2shha:gfp *in [26,67]) and subsequent blunting and relegating. The *0.8shha:gfp:z-shha-I2*, *0.8shha:gfp:z-shha-arC*, and *0.8shha:gfp z-shhb-I2 *were created by subcloning the respective *Not*I/*Kpn*I fragments from *2.4shha:gfp:C *[62], *2.4shha:gfp:ΔC*, and *2.4shha:gfp:shhb C *(Müller and coworkers, unpublished data) into *0.8shha:gfp *plasmid. The plasmids *0.8shha:gfp:t-shha-I2 *and *0.8shha:gfp:shhb-I2 *were made by reamplifying the respective intron 2 fragments from *pCRII-TOPO:t-shha-I2 *and *pCRII-TOPO:t-shhb-I2*, and subcloning them in *0.8shha:gfp *using *Not*I/*Kpn*I restriction sites. The *0.8shha:gfp:l-shh-I2 *was constructed by reamplifying the intron 2 part from the correct PCR fragment isolated from the BAC clone and cloning it into *0.8shha:gfp *(*Not*I/*Kpn*I). The *0.8shha:gfp:c-shh-I2 *and *0.8shha:gfp:m-shh-I2 *palsmids were made by direct cloning of the respective intron 2 fragments, amplified from genomic DNA, into *0.8shha:gfp *(*Not*I/*Kpn*I). The *0.8shha:gfp:z-shhb-non-cons *and *0.8shha:gfp:z-shhb-arC *were made by cloning the PCR-amplified nonconserved 5' part of z-*shhb I2 *(1032 bp) and the 380 bp 3' part containing the conserved region (*ar-C*) into *0.8shha:gfp *(*Not*I/*Kpn*I). All plasmids (*0.8shha:gfp:z-shha-arCΔ*C1, *0.8shha:gfp:z-shha-arCΔ*C2, *0.8shha:gfp:z-shha-arCΔ*C3; *0.8shha:gfp:z-shha-arCΔ*C4, *0.8shha:gfp:z-shha-arC+*C4m, *0.8shha:gfp:z-shhb-arCΔ*C1, and *0.8shha:gfp:z-shhb-arCΔ*C3) containing *z-shha-ar-C *or z-*shhb ar-C *carrying mutations in one of the conserved motifs (C1 to C4) were created by replacing the respective wild-type sequence of each conserved block with random sequence using a PCR-based approach. The same method was used to introduce the C2 and C4 from z-*shha ar-C *or random sequence into z-*shhb ar-C *(*0.8shha:gfp:z-shhb-arC+*C2, *0.8shha:gfp:z-shhb-arC+*C4, *0.8shha:gfp:z-shhb-arC+*C2rnd, and *0.8shha:gfp:z-shhb-arC+*C4rnd). The PCR products were cloned into *0.8shha:gfp *(*Not*I/*Kpn*I) and verified by sequencing.

The zebrafish *shha ar-C *[26] and *shhb ar-C *sequences can be found in GenBank under the following accession numbers: AL929206 (gi|34221785|, emb|AL929206.6|, region: 111,511 to 111,717 bp) for the *shha ar-C *and BX510360 (gi|46518135|, emb|BX510360.8|, region: 88,241 to 88,620 bp) for the *shhb ar-C*. The GenBank accession numbers for tench *shha *and *shhb *and *Latimeria shh *intron 2 sequences are as follows: EF593170, EF593171, and EF593172. For more detailed information about the sequences, which have been mutated and introduced in *shha *and *shhb *ar-Cs, see Table 2. The plasmid *2.7shhb:gfp *was constructed by replacing the *2.4shha *promoter fragment (*Sal*I/*Xho*I) from *2.4shha:gfp *with the PCR-amplified 2.7 kb *shhb *promoter fragment (upstream from the translation start site). The plasmid *2.7shhb:gfp:z-shhb-I1 *and *2.7shhb:gfp:z-shhb-I2 *were made by subcloning the *shhb I1 *and *I2 *from *2.4shha:gfp:shhb-I1 *and *2.4shha:gfp:shhb-I2 *(Müller and coworkers, unpublished data) into *2.7shhb:gfp *(*Not*I/*Kpn*I). For sequence information on the oligonucleotides that were used, see Table 3. More detailed information about the plasmid constructions is available upon request.

**Table 2:** Sequences used to replace wild-type sequence in *shha *and *shhb ar-C*s to generate the specified reporter constructs

Construct name	Wild-type sequence	Mutated/introduced sequence
*0.8shha:gfp:z-shha-arCΔ*C1	TGCACCTGAGCAAATA	GTACAAGTCTACCCGT
*0.8shha:gfp:z-shha-arCΔ*C2	GAAGTGTCCTTTTCCAAGAGT	TCCTGTAAGCCCAAGCTCTAC
*0.8shha:gfp:z-shha-arCΔ*C3	AATGACAATGTCC	CCGTCACCGTGAA
*0.8shha:gfp:z-shha-arCΔ*C4	CTTTATTGGTTTTTAATTAGA	AGGGCGGTTGGGGGCAGGCGG
*0.8shha:gfp:z-shha-arC+*C4m	CTTTATTGGTTTTTAATTAGA	CTTTATTGAGTTTTTTTAAATTAAGG
*0.8shha:gfp:z-shhb-arCΔ*C1	TGCACCTGTGTAAACA	GTACAAGTCTACCCGT
*0.8shha:gfp:z-shhb-arCΔ*C3	TTTAAATGACAATGTCT	GGCTCCGTCACCGTGAA
*0.8shha:gfp:z-shhb-arC*+C2	CAGGGAAAAGCACAGTCTGT	GAAGTGTCCTTTTCCAAGAGT
*0.8shha:gfp:z-shhb-arC*+C4	GACTTTGTGTAAATTCAGCAG	CTTTATTGGTTTTTAATTAGA
*0.8shha:gfp:z-shhb arC+*C2rnd	CAGGGAAAAGCACAGTCTGT	TCTCCAGGCTCAACCATGAGC
*0.8shha:gfp:z-shhb-arC+*C4rnd	GACTTTGTGTAAATTCAGCAG	AGAAAGCTCGCGCGACCATGA

**Table 3:** Primer sequences used for the amplification of the specified fragments

Sequence name	Forward primer	Reverse primer
Tench *shha *intron 2	GCIGGITTYGACTGGGTCTA (degenerative, used for isolation)	GAGTACCAGTGSAYICCIKC (degenerative, used for isolation)
Tench *shha *intron 2	GTAAGACCATGGCAGGATG (specific, used for subcloning)	TCGAGATAATAGCAATGGGT (specific, used for subcloning)
Tench *shhb *intron 2	GCIGGITTYGACTGGGTCTA (degenerative, used for isolation)	GAGTACCAGTGSAYICCIKC (degenerative, used for isolation)
Tench *shhb *intron 2	GTGAGAGCAATGTCACC (specific, used for subcloning)	GCGATAAAAGTAAAAAGAGAC (specific, used for subcloning)
*Latimeria shh *intron 2	TCAAAGCAGGTAAGCAGACG	AAGCAACCCCCTGATTTTG
Mouse *shh *intron 2	GTGGAAGCAGGTTTCGACTG	GAAAGACCAGGTGTTGAGTGC
Chick *shh *intron 2	CGGCTTCGACTGGGTCTAC	GCTGCCACTGAGTTTTCTGC
Zebrafish *shhb ar-C*	CCGAATAACAACAACTCGCAATC	CTGAGAAGATATACAAACACAA
Zebrafish *shhb *intron 2, nonconserved part	GTGAGCAAAAGCTGATATGC	GATTGCGAGTTGTTGTTATTCGG
2.7 kb zebrafish *shhb*, promoter	CATCTAAATCAACTGCAAGAACG	GACGTTTGAATTATCTCTTCTGGTC

In the degenerative oligonucleotides, in which the occurrence of all four nucleotides was equally possible, an inosine (I) was introduced to reduce degeneracy. On all specific primers, restriction enzyme sites were added (see Materials and methods for details).

### Microinjection and expression analysis

All microinjection experiments were performed with injection solution containing circular plasmid at a concentration of 10 to 15 ng/μl, supplemented with 0.1% phenol red. The solution was injected trough the chorion into the cytoplasm of zygotes. The GFP expression was analyzed on 24-hour-old embryos using Leica MZ FLIII fluorescent stereomicroscope (Leica Microsystems GmbH, Wetzlar, Germany). The level of expression was quantified by counting the number of GFP-positive cells in notochord and floor plate, as well as the number of ectopic GFP-positive cells in tissues where *shh(a) *and *shhb *are normally not expressed.

### Sequence alignments and analysis

Pair-wise sequence aliments were performed using one of the global alignment algorithms, namely AVID [97] in the case of intronic sequences (Figures 2a and 3a) and Shuffle-Lagan [98] in case of the whole *hh *loci (Figure 1), and visualized using Vista [99,100].

The multiple alignment of the intronic sequences was made using two algorithms, namely CHAOS/DIALIGN [71] or MUSCLE [101,102], and visualized using BioEdit (sequence alignment editor written by Tom Hall, Ibis Therapeutics, Carlsbad, CA, USA).

## Additional data files

The following additional data are available with the online version of this paper. Additional data file 1 provides a comparison of the expression pattern between stable transgenic lines and transient transgenic embryos. Additional data file 2 provides a synteny comparison of *shha *and *shhb *containing chromosomes, which suggests the loss of a duplicated *shh *paralog gene in medaka. Additional data file 3 shows multiple sequence alignment of *ar-C *enhancer homolog sequences from several vertebrate species.

## Supplementary Material

Additional data file 1 (A) Stable transgenic line (left) and transient transgenic embryos generated with gfp reporter construct, containing the 2.4 kb (sequence 2.4 kb upstream from the transcriptional start site) zebrafish *shha promoter*. (B to E) Transgenic lines and transient transgenic embryos generated with reporter constructs containing the 2.4 kbzebrafish *shha *promoter and zebrafish *ar-C *enhancer (B), tench *shha *intron 2 (C), *Latimeria shh *intron 2 (D) and zebrafish *shhb *intron 2. The numbers on the right side of the images of the stable transgenic lines indicate the number of the transgenic lines showing the expression pattern/total number of stable lines generated. ar, activation region; fp, floor plate; I, intron; l, *Latimeria*; nt, notochord; pr, promoter; t, tench; z, zebrafish. Click here for file

Additional data file 2 Shown are Ensembl views of zebrafish chromosome 7, containing the *shha *locus alongside medaka chromosome 20 (A), and zebrafish chromosome 2, containing the *shhb *locus alongside medaka chromosome 17 (B). Click here for file

Additional data file 3 Multiple sequence alignment of *ar-C *enhancer homolog sequences from several vertebrate species, performed with two alignment-algorithms, CHAOS-DIALIGN (A) and MUSCLE (B), reveals specific changes in the conserved putative transcription factor binding sites 2 and 4 (C2 and C4) of acanthopterygian fishes, which lack a *sonic hedgehog b *gene (for instance, medaka, stickleback, and pufferfish), as compared with ostaryophisian fishes, which have *sonic hedgehog b *(for example, zebrafish, tench, and Mexiacan cavefish [*Astyanax mexicanus*]). The C2 and C4 sites are marked with blue frames, and the differences in the C2 and C4 sequences in the acanthopterygian fishes are highlighted in yellow and orange, respectively. Click here for file
