# Supplementary figures and images for: Functional diversification of sonic hedgehog paralog enhancers identified by phylogenomic reconstruction
Source: Genome Biol. 2007 Jun 8;8(6):R106. doi: 10.1186/gb-2007-8-6-r106 (PMC2394741; doi:10.1186/gb-2007-8-6-r106)

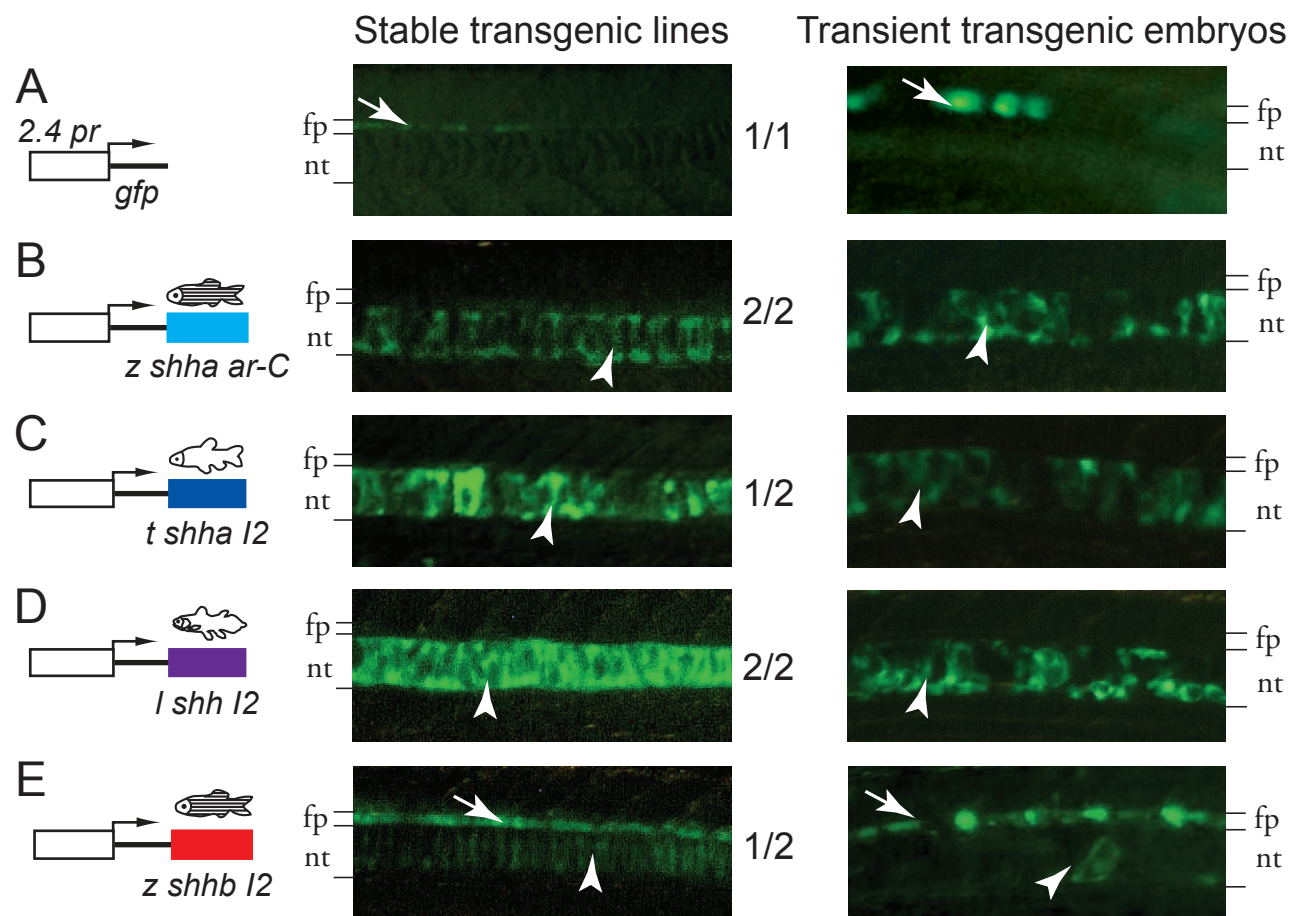

Supplement: Additional data file 1 — (A) Stable transgenic line (left) and transient transgenic embryos generated with gfp reporter construct, containing the 2.4 kb (sequence 2.4 kb upstream from the transcriptional start site) zebrafish shha promoter. (B to E) Transgenic lines and transient transgenic embryos generated with reporter constructs containing the 2.4 kbzebrafish shha promoter and zebrafish ar-C enhancer (B), tench shha intron 2 (C), Latimeria shh intron 2 (D) and zebrafish shhb intron 2. The numbers on the right side of the images of the stable transgenic lines indicate the number of the transgenic lines showing the expression pattern/total number of stable lines generated. ar, activation region; fp, floor plate; I, intron; l, Latimeria; nt, notochord; pr, promoter; t, tench; z, zebrafish. [file gb-2007-8-6-r106-S1.pdf]

A

Zebrafish  
Chr.7

Medaka  
Chr.20

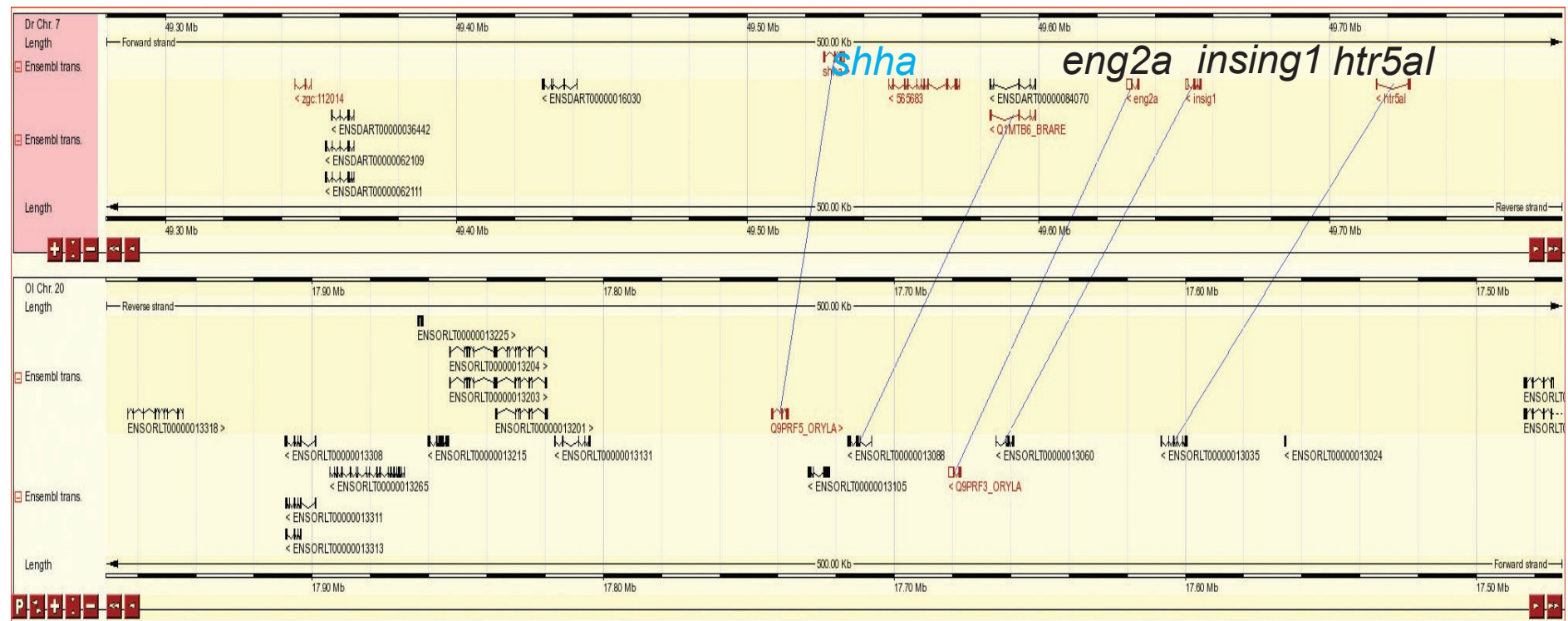

B

Zebrafish  
Chr.2

Medaka  
Chr.17

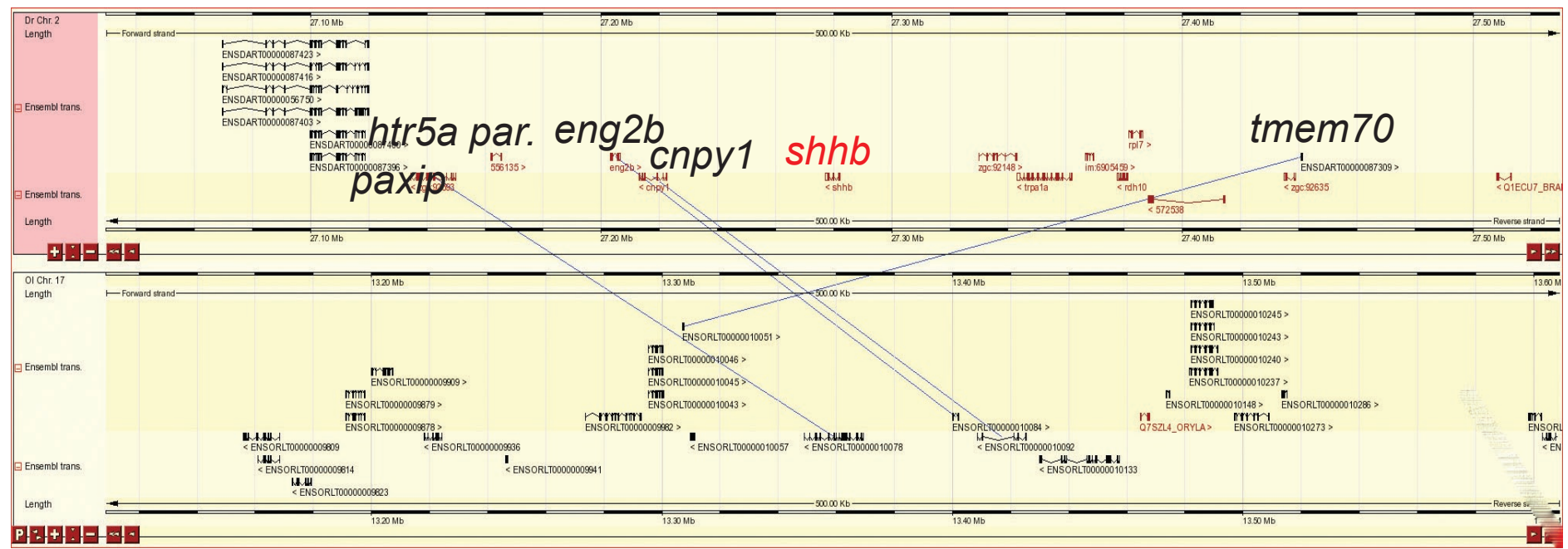

Supplement: Additional data file 2 — Shown are Ensembl views of zebrafish chromosome 7, containing the shha locus alongside medaka chromosome 20 (A), and zebrafish chromosome 2, containing the shhb locus alongside medaka chromosome 17 (B). [file gb-2007-8-6-r106-S2.pdf]

A

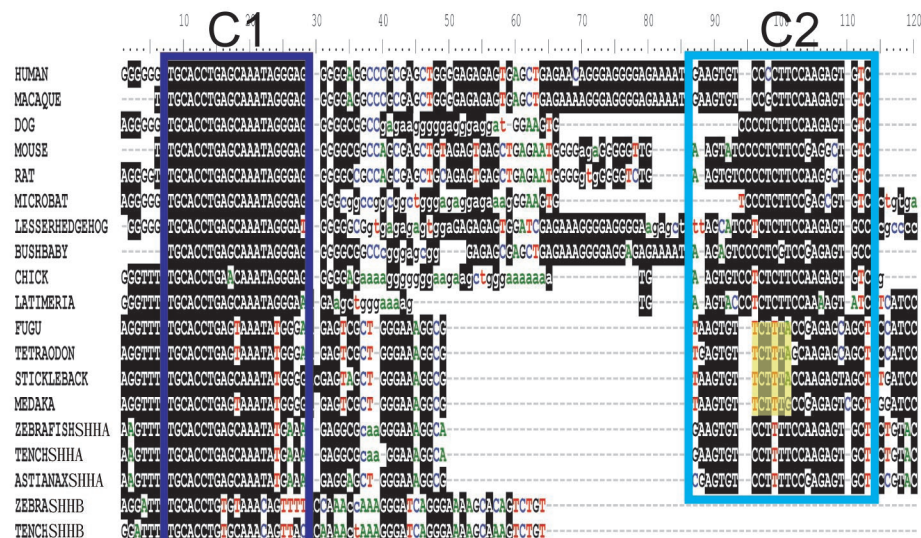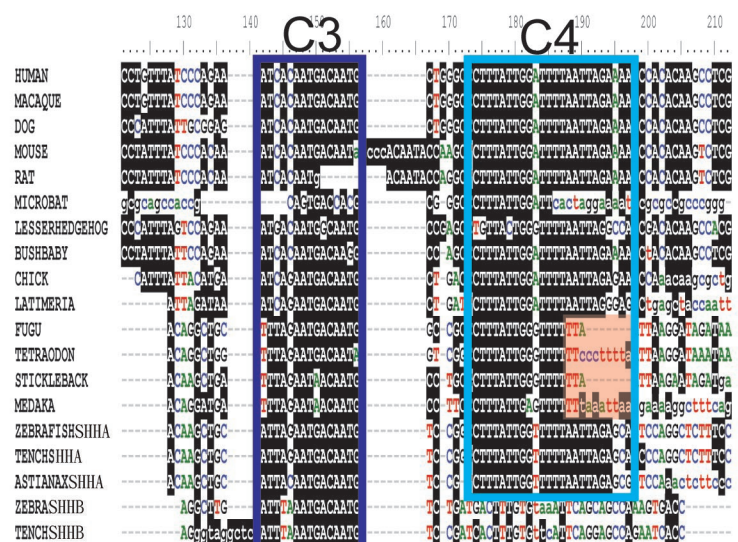

B

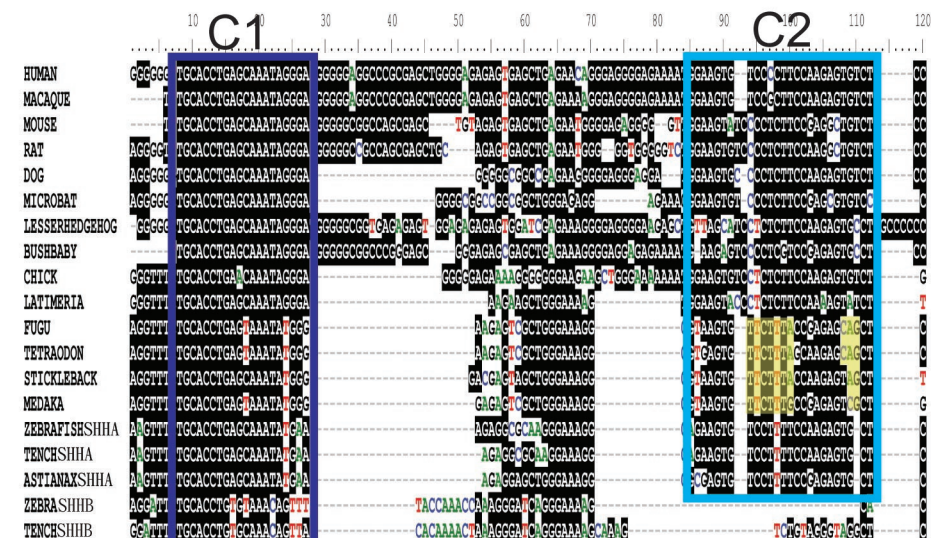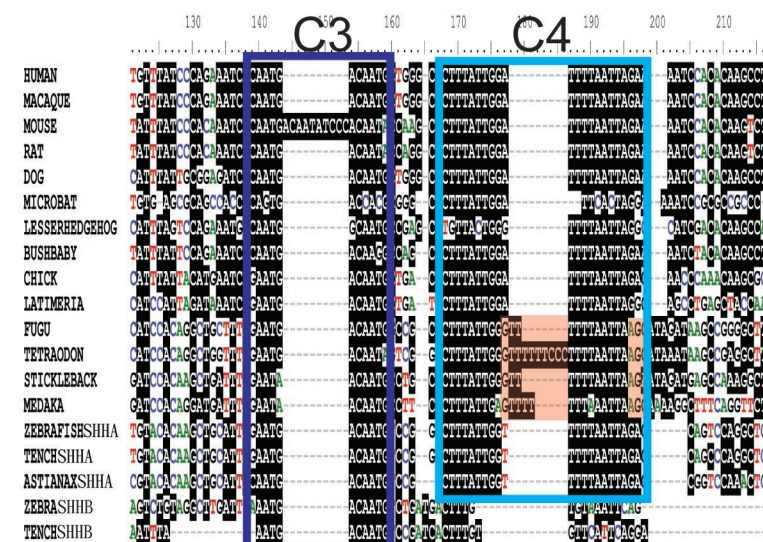

Supplement: Additional data file 3 — Multiple sequence alignment of ar-C enhancer homolog sequences from several vertebrate species, performed with two alignment-algorithms, CHAOS-DIALIGN (A) and MUSCLE (B), reveals specific changes in the conserved putative transcription factor binding sites 2 and 4 (C2 and C4) of acanthopterygian fishes, which lack a sonic hedgehog b gene (for instance, medaka, stickleback, and pufferfish), as compared with ostaryophisian fishes, which have sonic hedgehog b (for example, zebrafish, tench, and Mexiacan cavefish [Astyanax mexicanus]). The C2 and C4 sites are marked with blue frames, and the differences in the C2 and C4 sequences in the acanthopterygian fishes are highlighted in yellow and orange, respectively. [file gb-2007-8-6-r106-S3.pdf]
